# Analyzing Positional and Temporal Variations in Worst-Case Scenario Demands in Professional Spanish Soccer

**DOI:** 10.3390/jfmk10020172

**Published:** 2025-05-13

**Authors:** David Lobo-Triviño, Tomás García-Calvo, Jorge Polo-Tejada, Borja Sanabria-Pino, Roberto López del Campo, Fabio Nevado-Garrosa, Javier Raya-González

**Affiliations:** 1 Faculty of Sport Sciences, University of Extremadura, 10003 Caceres, Spain; davidlt@unex.es (D.L.-T.); jpolotej99@gmail.com (J.P.-T.); bsanabriapino@gmail.com (B.S.-P.); 2 Deparment of Competitions and Mediacoach, LaLiga, 28043 Madrid, Spain; rlopez@laliga.es (R.L.d.C.); fnevado@laliga.es (F.N.-G.); 3 Research Group on Sport and Physical Education for Personal and Social Development (GIDEPSO), Department of Specific Didactics, Faculty of Education Sciences and Psychology, University of Córdoba, 14004 Córdoba, Spain; rayagonzalezjavier@gmail.com

**Keywords:** match analysis, physical performance, football, tracking system, most demanding passages

## Abstract

Objectives: This study aimed to compare the worst-case scenario (WCS; i.e., 1-min) demands, in terms of distance covered and the number of times exceeding 85% of WCS demands, across soccer playing positions and match periods. Methods: A total of 67,518 records from 380 soccer matches during the 2023/24 season of the First Spanish Division were collected. Match events were tracked using the optical tracking system ChyronHego^®^ (TRACAB Gen5, NY, USA) and synchronized with Mediacoach software (LaLiga, Madrid, Spain). Total distance (TD), very high-speed running (VHSR), and sprint distance were considered, and all analyses were performed using linear mixed models (LMM). Results: Center-backs (CBs) exhibited significant differences (*p* < 0.001) in TD compared to all other positions. Regarding actions exceeding 85% of the 1-min WCS for TD, full-backs (FBs) showed significant differences compared to CBs (*p* < 0.001), midfielders (MDs; *p* < 0.001), and attacking midfielders (AMs; *p* < 0.001). In terms of VHSR, significant differences were observed between MDs and wingers (Ws; *p* < 0.05). In relation to match periods, during 15–30, CBs demonstrated significant differences compared to all other positions for actions exceeding 85% of the 1-min WCS in TD. Conclusions: These findings suggest that training protocols could be adjusted to account for specific positional demands, particularly focusing on high-speed running and sprint actions.

## 1. Introduction

Understanding the physical demands of competition is essential for preparing athletes to handle critical in-game situations effectively [1]. Match running performance in soccer, both in terms of total distance and at different speeds, considering different situational factors [2], age [3], or other variables, has been extensively studied for match analysis. However, considering the total match activity does not take the natural intermittence of the game into account [4]. As a result, various studies have introduced concepts to identify peak match demands. For instance, a study by Martín-García, Casamichana, Díaz et al. [5] analyzed the most demanding passages of play in professional soccer, focusing on high-intensity activities during matches. Similarly, research by Delaney et al. [6] examined peak running intensity periods in elite rugby, which, while focusing on a different sport, provides insights into methodologies applicable to soccer. However, perhaps the most commonly used concept to delve into the peak demands encountered by professional soccer players during competition is worst-case scenarios (WCS) [7]. The analysis of WCS, defined as the most intense periods (e.g., 1, 3, or 5 min) during a match or training session [1], has been increasingly investigated [8]. For example, Riboli et al. [9] showed significant differences in 1-min WCS between playing positions in the Italian Serie A. Therefore, it is important for the technical staff to know the nature of the WCS so their training approach can optimize the players’ performance [10] and reduce the injury risk [7].

Although the WCS is a relatively new concept, previous studies have explored its relationship with different variables [8]. Technological tools, such as Global Positioning Systems (GPS; Trewin et al., [11]) and optical tracking systems [7], facilitate the monitoring of players and the extraction of detailed information about WCS. For instance, in terms of total distance, the WCS for players in the reserve squad of a Spanish LaLiga team was reported to be 179 m·min^−1^ [12], whereas for an English Championship team, it was 190 m·min^−1^ [13]. The most demanding passages of WCS have also been studied in high-intensity terms. In the Spanish Second Division, Oliva-Lozano, Fortes, & M. Muyor [14] showed that 1-min WCS for high-speed running distance (>21 km·h^−1^) was 61 m·min^−1^, while for sprint distance (>24 km·h^−1^) it was 30 m·min^−1^. However, the studies conducted so far have been limited by a small sample involving players from a single team and analyzed across only a few matches. Consequently, further research with larger sample sizes and data collected over an entire season is warranted.

To optimize the aforementioned aims, it is relevant to consider certain contextual variables that influence the WCS demands during the matches, with particular emphasis on playing positions [10,15]. In this regard, Oliva-Lozano et al. [1] observed significant differences in 1-min WCS between central defenders and midfielders in terms of total distance. For sprint distance, forwards and central defenders presented significant differences in 1-min WCS with respect to wide midfielders (37.7 m·min^−1^ and 35.6 m·min^−1^ vs. 48.5 m·min^−1^, respectively; Riboli et al. [9]). Another contextual variable with a key role in soccer demands is the match period. Some studies have reported that the match running performance of soccer players can vary over match time, showing a decrease in player activity in the second halves [12]. Specifically, for total distance, the 1-min WCS of the midfielders of the reserve squad of a Spanish LaLiga team was higher in the first half than in the second half (196.4 m·min^−1^ vs. 190.2 m·min^−1^). Similarly, Oliva-Lozano et al. [1] showed that the total distance covered in the 1-, 3-, 5-, and 10-min WCSs was significantly higher in the first half. However, no significant differences were found for the high-speed running distance and sprint distance WCS. These insights highlight the importance of considering playing position and time periods when analyzing the WCS, which allows for a more comprehensive understanding of the demands placed on players in different roles and different match moments.

Despite the increase in research focused on contextualized analysis of WCS in professional soccer, to our knowledge, only WCS in whole matches or differentiating between halves have been analyzed; no study has analyzed WCS differentiating by time periods. Moreover, previous studies have only considered a reduced number of matches and players, whereas we have included all the teams’ matches during a full season. Therefore, the aim of the study was to analyze the influence of playing positions and match period (15 min) on the 1-min WCS and on the number of times in which players achieved 85% of the 1-min WCS in the Spanish First Division during a full season. Based on previous studies [1,12], we hypothesized that time periods substantially influence the 1-min WCS and the number of times in which players achieved 85% of WCS, with differences between playing positions.

## 2. Materials and Methods

### 2.1. Study Design

A retrospective, descriptive longitudinal design was applied to examine the differences in 1-min WCS demands, in terms of distance covered at different intensities and the number of times in which players achieved 85% of 1-min WCS, among playing positions and time periods in professional Spanish soccer players. The 1-min WCS was selected to minimize the influence of technical-tactical situations and to ensure it is as representative as possible for practical purposes. Data on distance covered in the WCS and the number of times players exceeded 85% of the WCS were collected by Mediacoach, similar to previous studies using 85% maximum speeds and intensities [16]. In addition, soccer players were classified according to their position on the field into six groups: center back (CB), full back (FB), midfielder (MD), attacking midfielder (AM), winger (W), and striker (S). The matches were divided into 6 periods: 0–15 (time period from minute 0 to minute 15 of the match), 15–30 (time period from minute 15 to minute 30 of the match), 30–45 (time period from minute 30 to minute 45 of the match), 45–60 (time period from minute 45 to minute 60 of the match), 60–75 (time period from minute 60 to minute 75 of the match), and 75–90 (time period from minute 75 to minute 90 of the match).

### 2.2. Sample

A total of 380 matches of the 2023/24 season from the First Spanish Division were considered for the analysis. Thus, 67,518 individual match observations from 587 professional soccer players were included. Goalkeepers were not included in the analysis due to their specific role during the game. Data were provided to the authors by LaLiga^TM^ (Madrid, Spain), which informed all participants through its protocols. All data were anonymized according to the Declaration of Helsinki to ensure “player” and “team” confidentiality. The study was fully approved by the Ethics Committee of the University of Extremadura; Vice-Rectorate of Research, Transfer and Innovation—Delegation of the Bioethics and Biosafety Commission (Protocol number: 239/2019).

### 2.3. Variables and Procedure

The optical tracking system TRACAB (ChyronHego VID, New York, NY, USA) was used to collect the match running performance data. This multi-camera tracking system consists of eight super 4K High Dynamic Range cameras based on a positioning system (Tracab—ChyronHego VTS) which records and analyses X and Y positions of each player from several angles, providing real-time two-dimensional tracking with a sampling frequency of 25 Hz. Additionally, a customized report was created using Mediacoach software (LaLiga, Madrid, Spain), which synchronized tracking data with the video footage of each match. The validity and reliability of this system for the variables used have previously been investigated [17,18] and reported strong correlations (*r* > 0.80) and high intraclass correlation coefficients (*r* > 0.75) between the Mediacoach multicamera tracking system and Global Positioning System. WCS demands was divided into the following categories: total distance covered by player in meters during the 1-min WCS (TD); distance covered by player in meters above 21 km·h^−1^ during 1-min WCS (VHSR); distance covered by player in meters above 24 km·h^−1^ during 1-min WCS (Sprint); number of times in which a player has exceeded 85% of the 1-min WCS in terms of TD (TD_85%_); number of times in which a player has exceeded 85% of the 1-min WCS in terms of VHSR distance (VHSR_85%_); number of times in which a player has exceeded 85% of the 1-min WCS in terms of Sprint distance (Sprint_85%_).

### 2.4. Statistical Analysis

All statistical analyses were performed with the RStudio *lme4* package [19,20]. Given the hierarchical structure of the sample, with data nested in groups and exhibiting a longitudinal nature, linear mixed models (LMM) were deemed the most suitable analytical approach. LMM has been proven effective for handling unbalanced and repeated-measures data [21,22].

In this study, WCS demands during matches were nested within players, with each player having multiple observations across different matches and each match comprising data from several players. Furthermore, players were nested within different teams each season. As the data structure is not strictly hierarchical, cross-classified multilevel models were employed. This approach allowed the progressive inclusion of fixed and random effects following general multilevel modeling strategies [21].

LMM were used to analyze the effects of playing positions on player WCS demands. Initially, a two-level hierarchy was modeled. WCS demands (i.e., distances covered at different speed thresholds and the number of times where a player has exceeded 85% of the 1-min WCS) were included as dependent variables, while playing positions (i.e., center defender [CD], full-back [FB], midfielder [MD], attacking midfielder [AM], winger [W], and striker [S]) and match periods (i.e., 0–15, 15–30, 30–45, 45–60, 60–75, and 75–90 min) were the independent variables included as fixed effects. The soccer player variable was considered a random effect. Results were reported as coefficients and standard errors (Coeff ± SE), and statistical significance was set at *p* < 0.05.

## 3. Results

**Figure 1:**
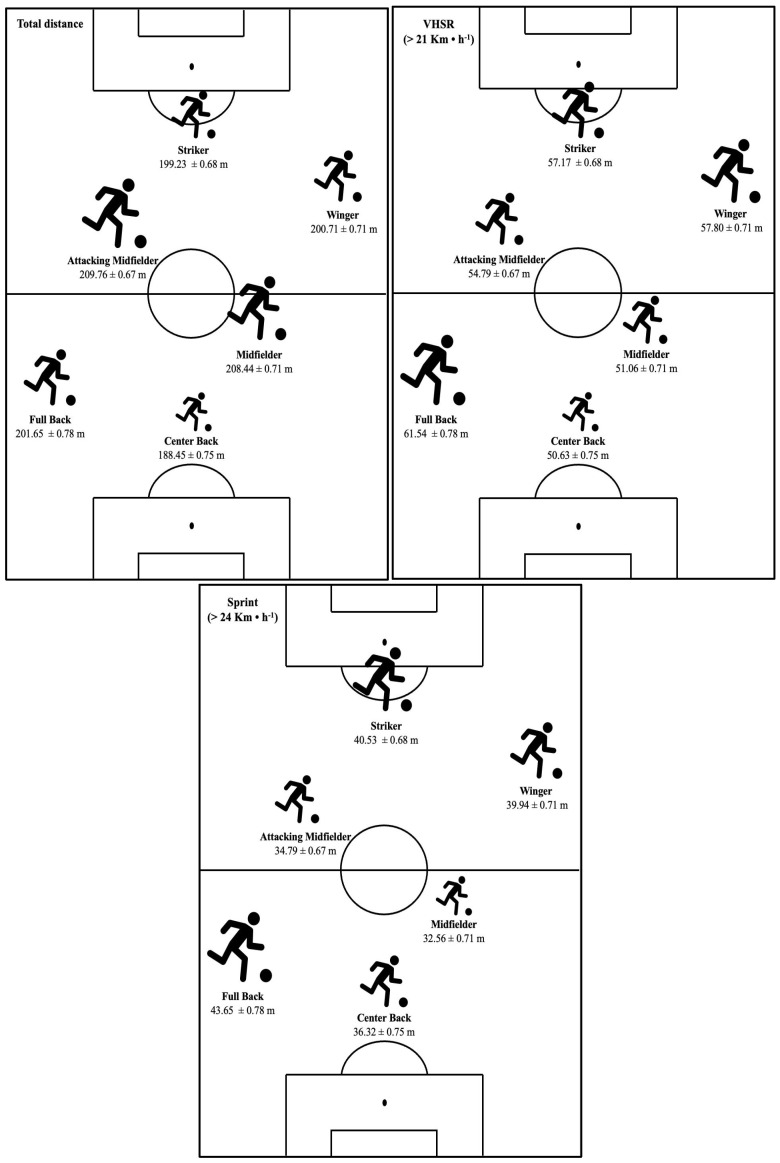
One-min WCS demands (in meters) according to playing positions.

Figure 1 shows 1-min WCS demands (in meters) according to playing positions. Regarding TD, FB presented significant differences with respect to CB (*p* < 0.001), MD (*p* < 0.001), AM (*p* < 0.001), and S (*p* < 0.05). Similarly, CB presented significant differences with all positions (*p* < 0.001). MD and AM presented significant differences with respect to W and S (*p* < 0.001). In terms of VHSR, FB presented significant differences with all positions (*p* < 0.001). CB and MD presented significant differences with respect to AM, W, and S (*p* < 0.001). In attacking positions, AM presented significant differences with respect to W (*p* < 0.001) and S (*p* < 0.01). In relation to Sprint distance, similarly to VHSR, FB presented significant differences with all positions (*p* < 0.001). CB presented significant differences with respect to MD, W, and S (*p* < 0.001). MD presented significant differences with respect to attacking positions, AM (*p* < 0.01), W (*p* < 0.001), and S (*p* < 0.001). Finally, AM presented significant differences with respect to W and S (*p* < 0.001).

The number of times in which players exceeded 85% of 1-min WCS according to playing positions is presented in Table 1. Regarding TD_85%_, FB presented significant differences with respect to CB (*p* < 0.001), MD (*p* < 0.001), and AM (*p* < 0.001). CB, MD, and AM presented significant differences with respect to W (*p* < 0.001) and S (*p* < 0.001). Concerning VHSR_85%_, FB showed significant differences with all positions, except with W. Also, significant differences were found between MD and W (*p* < 0.05). In terms of Sprint_85%_, FB showed significant differences with respect to MD (*p* < 0.01), AM (*p* < 0.05), and S (*p* < 0.05). Finally, significant differences were found between MD and W (*p <* 0.05).

**Figure 2:**
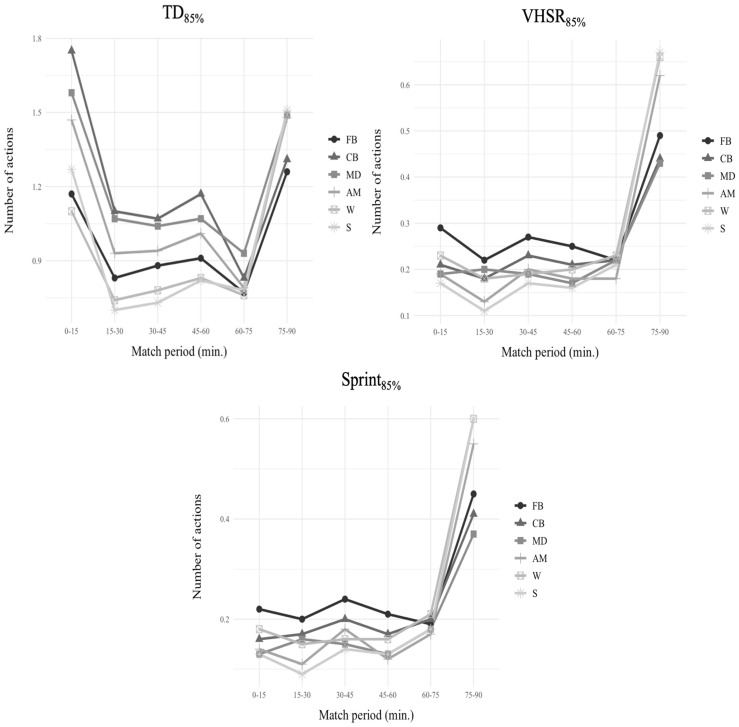
Number of times in which players achieved 85% of 1-min WCS according to playing position and match periods. FB = full back; CB = center back, MD = midfielder; AM = attacking Midfielder; W = winger; S = striker.

Figure 2 shows the number of times in which players exceeded 85% of 1-min WCS according to playing position and match periods.

Regarding TD_85%_, in the 0–15 period, FB showed significant differences with respect to CB (*p* < 0.001), MD (*p* < 0.001), AM (*p* < 0.001), and S (*p* < 0.05). CB presented significant differences compared to MD, AM, W, and S (*p* < 0.001). MD showed significant differences with respect to AM (*p* < 0.01), W (*p* < 0.001), and S (*p* < 0.001). AM presented significant differences compared to W and S (*p* < 0.001). Finally, W showed significant differences with respect to S (*p* < 0.001). During the 15–30 period, FB showed significant differences with respect to CB (*p* < 0.001), MD (*p* < 0.001), AM (*p* < 0.01), W (*p* < 0.01), and S (*p* < 0.001). CB and MD presented significant differences compared to AM, W, and S (*p* < 0.001). AM presented significant differences compared to W and S (*p* < 0.001). In the 30–45 period, FB showed significant differences with respect to CB (*p* < 0.001), MD (*p* < 0.001), W (*p* < 0.01), and S (*p* < 0.001). CB and MD presented significant differences compared to AM, W, and S (*p* < 0.001). AM showed significant differences with respect to W and S (*p* < 0.001). During the 45–60 period, FB showed significant differences with respect to CB (*p* < 0.001), MD (*p* < 0.001), AM (*p* < 0.01), W (*p* < 0.05), and S (*p* < 0.01). CB presented significant differences compared to MD (*p* < 0.01), AM (*p* < 0.001), W (*p* < 0.001), and S (*p* < 0.001). MD showed significant differences with respect to AM (*p* < 0.05), W (*p* < 0.001), and S (*p* < 0.001). AM presented significant differences compared to W and S (*p* < 0.001). In the 60–75 period, FB showed significant differences with respect to CB (*p* < 0.01) and MD (*p* < 0.001). CB presented significant differences compared to MD (*p* < 0.001) and W (*p* < 0.01). MD showed significant differences with respect to AM, W, and S (*p* < 0.001). During the 75–90 period, FB and CB presented significant differences compared to MD, AM, W, and S (*p* < 0.001).

Analyzing VHSR_85%_, FB showed significant differences with respect to MD (*p* < 0.05), AM (*p* < 0.05), and S (*p* < 0.001) during the 0–15 period. In the 15–30 period, FB presented significant differences compared to AM (*p* < 0.01) and S (*p* < 0.001). FB and W showed significant differences with respect to S (*p* < 0.05). MD presented significant differences compared to AM (*p* < 0.05) and S (*p* < 0.01). During the 30–45 period, FB showed significant differences with respect to MD (*p* < 0.05), AM (*p* < 0.05), W (*p* < 0.05), and S (*p* < 0.01). In the 45–60 period, FB presented significant differences compared to MD (*p* < 0.05), AM (*p* < 0.05), and S (*p* < 0.01). During the 75–90 period, FB showed significant differences with respect to AM (*p* < 0.05), W (*p* < 0.01), and S (*p* < 0.001). Similarly, CB and MD presented significant differences compared to AM, W, and S (*p* < 0.001).

Finally, concerning Sprint_85%_, in the 0–15 period, FB showed significant differences with respect to MD and S (*p* < 0.05). During the 15–30 period, FB presented significant differences compared to AM (*p* < 0.01) and S (*p* < 0.001). CB and MD showed significant differences with respect to S (*p* < 0.05). In the 30–45 period, FB presented significant differences compared to MD (*p* < 0.01), W (*p* < 0.05), and S (*p* < 0.01). CB showed significant differences with respect to S (*p* < 0.05). During the 45–60 period, FB presented significant differences compared to MD (*p* < 0.05), AM (*p* < 0.01), and S (*p* < 0.01). In the 75–90 period, FB showed significant differences with respect to AM (*p* < 0.05), W (*p* < 0.01), and S (*p* < 0.01). Similarly, CB and MD presented significant differences compared to AM, W, and S (*p* < 0.001).

## 4. Discussion

This study aimed to compare the WCS demands, in terms of distance covered and the number of times a player has exceeded 85% of the 1-min WCS, among playing positions and time periods in professional Spanish soccer players. This is the first study that analyzes the number of actions in WCS, considering all teams, during a whole season in a professional soccer league, and differentiating among match periods, providing extensive information that could serve as a robust reference for the scientific community and practitioners. In all velocity ranges, FB players covered the greatest WCS distance and achieved a greater number of times exceeding 85% of the 1-min WCS compared to their counterparts. Additionally, at high intensity, W and S exhibited higher values in their WCS (in meters) and achieved a greater number of times exceeding 85% of the 1-min WCS compared to CB, MD, and AM. These results support the hypotheses previously formulated.

Given that previous studies have highlighted the differentiated demands in soccer according to playing positions [23,24,25], including sprint variables [16], it is crucial to analyze whether WCS vary based on these positions. This would allow for the adequate dosage of training loads to optimize the players’ performance and, more relevantly, reduce the risk of injury [26]. Consistent with the findings of Martín-García, Casamichana, Díaz, et al. [5], where MD players exhibited the greatest TD in their 1-min WCS, our study revealed significant differences between playing positions. Specifically, MD and AM recorded the highest values compared to their counterparts. When examining high-intensity, with velocities greater than 21 km·h^−1^, FB recorded the greatest distances covered, showing significant differences compared to all other playing positions. These findings align with previous research [5,14]. W and S, in turn, showed significant differences with CB, MD, and AM in the same variable. This could be due to the fact that wide players (i.e., FB and W) and S have higher high-speed running demands than other positions, necessitating more meters in their WCS. Additionally, when the velocity threshold was raised to above 24 km·h^−1^, FB again recorded the highest distance values, showing significant differences from all other positions. In addition, CB covered more meters than MD but fewer than W, who also covered more meters than MD. Furthermore, W and S completed more meters sprinting in their WCS compared to CB, MD, and AM. These results are consistent with previous studies [5,14], which reported that FB had the highest values of meters covered in their WCS during sprint actions. Practically, these results suggest the need to increase densities when specifically training the WCS for FB W and S players, particularly in high-speed running and sprinting.

To enhance the effectiveness of the training process, it is essential to focus on individualization and well-structured task design, considering not only the distances covered in each WCS but also the number of times in which players exceeded 85% of 1-min WCS and how these variables vary across playing positions. In our study, CB and MD performed a greater number of TD_85%_. However, when considering high-speed running (i.e., distance covered > 21 km·h^−1^, VHSR_85%_), FB presented significantly higher values than the rest of their counterparts, except for W. This is because the tactical functions of full backs involve a high number of high-intensity actions both in attack and defense [27]. On the other hand, W performed more VHSR_85%_ compared to MD, who, despite performing long actions, are not characterized by the repetition of high-intensity efforts [16,28]. When considering distances above 24 km·h^−1^ (Sprint_85%_), the results are similar to those reported for the 21 km·h^−1^ range, with FB completing a greater number of times than MD, AM, and S, while the latter two positions presented higher values than MD. The findings obtained in this study provide valuable information for the comprehensive design of soccer training tasks, with a special focus on the WCS.

Finally, to optimize training periodization based on the individualized demands of soccer players, it is essential to understand how WCS are distributed throughout a match for each playing position. Regarding TD_85%_, in all match periods, CB and MD consistently presented higher values, except for the 75–90 min interval, during which S exhibited the greatest number of times exceeding 85% of WCS. This trend may be explained by the fact that CB and MD typically cover fewer meters at high intensities compared to other positions. Consequently, by not engaging in as many intense efforts, these players are able to accumulate greater total distances [27]. Additionally, the first and final periods of the match were characterized by the highest number of WCS actions across all playing positions. Focusing on workload characteristics related to VHSR_85%_ and Sprint_85%_, FB demonstrated the highest number of times during all match periods except the 75–90 min interval. In this final period, AM, W, and S (i.e., positions located closer to the opponent’s goal) exhibited a greater number of times exceeding 85% of WCS. This can be attributed to increased efforts to score decisive goals as the match nears its conclusion, with these actions predominantly involving sprints [29]. Furthermore, this period is the most physically demanding for all positions, as it often determines the outcome of the match [27]. These positional and temporal differences in WCS must be carefully considered by strength and conditioning coaches to tailor training programs more effectively. By accounting for the unique demands placed on each playing position, clubs can enhance player performance and ensure optimal preparation for competition.

This manuscript has some limitations that should be acknowledged. Firstly, all matches were considered together without accounting for other variables that can influence external demands (increasing or decreasing WCS meters), such as effective playing time, ball possession, playing styles, or match status. Secondly, only male players were included in the study, which may limit the extrapolation of the results to the female population. Thirdly, the 3- and 5-min WCSs were not considered in the analysis, although they could complicate the results and their practical applications. Fourth, all players were considered, with no distinction between starters and substitutes. Finally, other relevant external load variables, such as accelerations and decelerations, were not considered. For these reasons, future studies addressing these limitations are recommended.

## 5. Conclusions

The present study explains and compares the differences in WCS and the number of times players exceeded 85% of 1-min WCS between playing positions and different match periods. WCS seems to be a relevant match performance variable that differs between positions and match periods. FB covered the most distance and executed the highest number of times exceeding 85% of 1-min WCS, when high-intensity and sprint variables are considered, compared to other positions. Moreover, W and S recorded higher WCS values (both in meters and times exceeding 85% of WCS) compared to CB, MD, and AM. Finally, in terms of VHSR_85%_ and Sprint_85%_, FB achieve higher values in all periods of the match, except for 75–90, where they are outperformed by the attacking players (AM, W, and S). In terms of practice, strength, and conditioning, practitioners should consider adapting their periodization to specific WCS scenarios. Specifically, it is recommended to increase training densities for FB, W, and S, especially focusing on high-speed running and sprint actions.

## Figures and Tables

**Table 1:** Number of times in which players achieved 85% of 1-min WCS according to playing position.

Variables	FB		CB		MD		AM		W		S	
	Coeff (SE)	*p*	Coeff (SE)	*p*	Coeff (SE)	*p*	Coeff (SE)	*p*	Coeff (SE)	*p*	Coeff (SE)	*p*
TD_85%_ (nº)	5.79 (0.09)	b ***, c ***, d ***	7.23 (0.07)	d ***, e ***, f ***	7.22 (0.07)	d ***, e ***, f ***	6.59 (0.07)	e ***, f ***	5.66 (0.07)		5.81 (0.07)	
VHSR_85%_ (nº)	1.70 (0.07)	b *, c **, d *, f *	1.50 (0.07)		1.42 (0.07)	e *	1.47 (0.07)		1.65 (0.07)		1.48 (0.07)	
Sprint_85%_ (nº)	1.49 (0.07)	c **, d *, f *	1.31 (0.07)		1.17 (0.07)	e *	1.26 (0.07)		1.40 (0.07)		1.26 (0.07)	

Note. Coeff = coefficient; SE = standard error; nº = number of actions; FB = full back; CB = center back; MD = midfielder; AM = attacking midfielder; W = winger; S = striker; TD_85%_ = number of times in which players achieved 85% of 1-min WCS in terms of TD; VHSR_85%_ = number of times in which players achieved 85% of 1-min WCS in terms of VHSR distance; Sprint_85%_ = number of times in which players achieved 85% of 1-min WCS in terms of Sprint distance; a = significant differences compared to FB; b = significant differences compared to CB; c = significant differences compared to MD; d = significant differences compared to AM; e = significant differences compared to W; f = significant differences compared to S; * *p* < 0.05; ** *p* < 0.01; *** *p* < 0.001.

## Data Availability

Restrictions apply to the availability of these data. Data were obtained from LaLiga and are available at https://www.laliga.es/en (accessed on 10 July 2024) with the permission of LaLiga.
